# Neutrophil microvesicles resolve gout by inhibiting C5a-mediated priming of the inflammasome

**DOI:** 10.1136/annrheumdis-2015-207338

**Published:** 2015-08-05

**Authors:** Arun Cumpelik, Barbara Ankli, Daniel Zecher, Jürg A Schifferli

**Affiliations:** 1 Department of Biomedicine, University Hospital Basel, Basel, Switzerland; 2 Department of Rheumatology, University Hospital Basel, Basel, Switzerland; 3 Department of Medicine, University Hospital Basel, Basel, Switzerland; 4 Department of Nephrology, University Hospital Regensburg, Regensburg, Germany

**Keywords:** Cytokines, Gout, Inflammation

## Abstract

**Objectives:**

Gout is a highly inflammatory but self-limiting joint disease induced by the precipitation of monosodium urate (MSU) crystals. While it is well established that inflammasome activation by MSU mediates acute inflammation, little is known about the mechanism controlling its spontaneous resolution. The aim of this study was to analyse the role of neutrophil-derived microvesicles (PMN-Ecto) in the resolution of acute gout.

**Methods:**

PMN-Ecto were studied in a murine model of MSU-induced peritonitis using C57BL/6, MerTK^−/−^ and C5aR^−/−^ mice. The peritoneal compartment was assessed for the number of infiltrating neutrophils (PMN), neutrophil microvesicles (PMN-Ecto), cytokines (interleukin-1β, TGFβ) and complement factors (C5a). Human PMN-Ecto were isolated from exudates of patients undergoing an acute gouty attack and functionally tested in vitro.

**Results:**

C5a generated after the injection of MSU primed the inflammasome for IL-1β release. Neutrophils infiltrating the peritoneum in response to C5a released phosphatidylserine (PS)-positive PMN-Ecto early on in the course of inflammation. These PMN-Ecto in turn suppressed C5a priming of the inflammasome and consequently inhibited IL-1β release and neutrophil influx. PMN-Ecto-mediated suppression required surface expression of the PS-receptor MerTK and could be reproduced using PS-expressing liposomes. In addition, ectosomes triggered the release of TGFβ independent of MerTK. TGFβ, however, was not sufficient to control acute MSU-driven inflammation in vivo. Finally, PMN-Ecto from joint aspirates of patients with gouty arthritis had similar anti-inflammatory properties.

**Conclusions:**

PMN-Ecto-mediated control of inflammasome-driven inflammation is a compelling concept of autoregulation initiated early on during PMN activation in gout.

## Introduction

Gout is a highly inflammatory arthritis induced by the precipitation of monosodium urate (MSU) crystals in articular joints. Even without intervention, acute gouty arthritis (GA) usually resolves spontaneously within a few days leaving minimal residual damage to the joint. What drives this timely resolution of gout, however, is not yet clear.

The early inflammatory phase of gout is characterised by the production of the proinflammatory cytokine interleukin-1β (IL-1β) and the infiltration of neutrophils into the joint space. IL-1β is the principle driving force of gouty inflammation and is released as a consequence of NLRP3 inflammasome assembly and caspase 1 activation.^1^ The underlying mechanism behind the resolution of gout must therefore interfere with the release of IL-1β.

Previous studies indicate that the generation of apoptotic leucocytes and their clearance by macrophages may play a key role in resolving gout.^2^ ^3^ The recognition of phosphatidylserine (PS) on the surface of apoptotic cells and the release of TGFβ upon their clearance are strong anti-inflammatory cues that can suppress an inflammatory response.^4^ PS-positive surfaces can engage the MerTK receptor initiating the transcription of suppressor of cytokine signalling (SOCS) 3.^5–7^ High levels of SOCS3 expression in synovial tissues as well as elevated levels of TGFβ in the synovial fluids of patients during the resolution phase of gout have been reported.^8^ Little is however known about how and where the inflammasome is regulated.

We previously established that human neutrophils stimulated by complement C5a or bacterial peptide fMLP (formyl-methionyl-leucyl-phenylalanine) in vitro release microvesicles (ectosomes) from their surface.^9^ These ectosomes express PS and are capable of inducing the release of TGFβ by monocyte-derived macrophages. In vitro, neutrophil-derived ectosomes have been shown to suppress the response to TLR ligands in monocyte-derived macrophages and dendritic cells.^10^

The aim of this study was to determine whether the anti-inflammatory effects of ectosomes extend to the NLRP3 inflammasome, whether ectosomes are found in vivo and whether they take part in the early resolution of gouty inflammation.

## Materials and methods

### Generation and analysis of PMN-Ecto and BM-Ecto

Mouse PMN-Ecto were derived from inflamed peritonea. The peritoneal cavity was lavaged with 5 mL PBS-1%FCS, 6–10 h after intraperitoneal injection of 3 mg MSU crystals, and the lavage fluid sequentially centrifuged to separate cells (350 g/10′/4°C), cell debris (3000 g/10′/4°C) and finally PMN-Ecto (50.000 g/40′/4°C). The final pellet was diluted in 0.2 µm filtered buffers and analysed by flow cytometry. PMN-Ecto were counted using Trucount beads (BD Biosciences, Allschwill, Switzerland) as follows: (% of total microvesicles/% of beads)×(absolute number of beads)×(% of annexin V^+^, Gr-1^+^ double positive events). Mouse bone marrow-derived ectosomes (BM-Ecto) were prepared from bone marrow cells obtained by flushing femurs and tibias of B6 mice. Erythrocytes were removed by hypotonic lysis and the remaining myeloid cells stimulated with 1 µM fMLP (Sigma, St Louis, Missouri, USA) or 10 ng/mL mouse recombinant C5a (BD Pharmingen) in RPMI for 30 min at 37°C. The supernatant was sequentially centrifuged as above. Where indicated, BM-Ecto were stained with 5 µM CFSE (Molecular Probes, Zug, Switzerland). Human PMN-Ecto were isolated by sequentially centrifuging joint exudates from patients with gout or control patients with osteoarthritis (OA). Flow cytometry was performed using a CyanADP cytometer (Beckman Coulter, Nyon, Switzerland). Data were analysed using FlowJo Software (TreeStar, San Jose, California, USA).

### Cell culture conditions, ELISA and western blot

Resident peritoneal macrophages were harvested by peritoneal lavage and plated at 2×10^6^/well. To achieve full inflammasome activation in vitro, cells were first primed with 10 ng/mL ultrapure LPS (Invivogen, Toulouse, France) or 10 ng/mL mouse recombinant C5a (BD) for 10 h, washed and subsequently stimulated with 100 µg/mL MSU. Macrophages were treated with BM-Ecto (1×10^8^ BM-Ecto/2×10^6^ macrophages) or liposomes containing either phosphatidylserine (PS) or control phosphatidylcholine (PC) (1×10^8^ liposomes/2×10^6^ macrophages) for 10 min either prior to LPS priming or prior to MSU stimulation. Cell extracts and cell culture supernatants were prepared for western blotting as described previously.^11^ SOCS3 protein expression was analysed by densitometry and normalised to actin using Image Lab software (Biorad, Munich, Germany).

Cell culture supernatants and peritoneal lavages were analysed for IL-1β, IL-10, IL-1Ra and TGFβ using the IL-1β, IL-10 OptEIA (BD Pharmingen), IL-1Ra (R&D, DY480) and TGFβ1 (eBiosciences, Vienna, Austria) ELISA kits, respectively, according to the manufacturer's instructions.

### Experimental peritonitis

Mice were treated with an intraperitoneal injection of 2×10^7^ BM- Ecto/PMN-Ecto or comparable amounts of PS or control PC liposomes 2 h prior to intraperitoneal stimulation with 3 mg MSU crystals. Control groups received Ecto followed by 0.9% NaCl or received the stimulus preceded by an injection of 0.9% NaCl instead of Ecto. At the indicated time points following induction of peritonitis, mice were sacrificed by CO_2_-inhalation and peritoneal lavage was performed with 5 mL PBS-1% FCS. The lavage fluid was filtered (70 μm), washed and contaminating red blood cells removed by hypotonic lysis. Leucocytes were counted with an automated cell counter (Beckman Coulter, Nyon, Switzerland). Peritoneal leucocytes were phenotyped by flow cytometry following surface staining with the indicated antibodies (see online supplementary figure S1A).

For detailed methods see online supplementary material.

## Results

### Bone marrow-derived ectosomes suppress inflammasome activation in vitro

**Figure 1:**
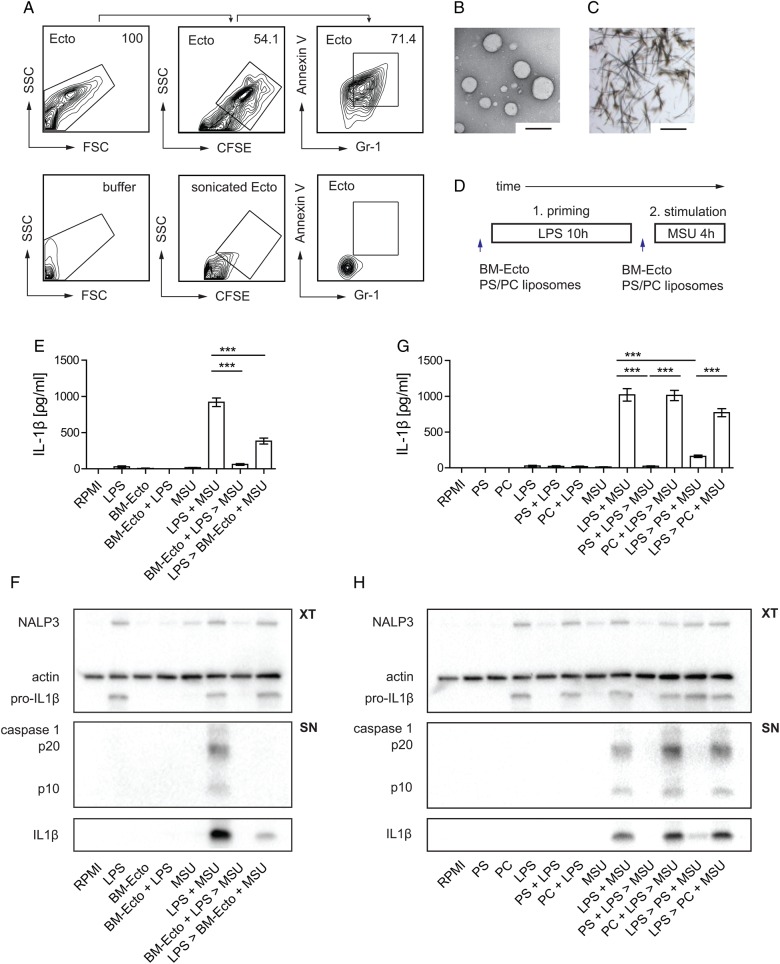
Characterisation and in vitro properties of BM-Ectosomes. (A) Flow cytometric characterisation of bone marrow-derived ectosomes (BM-Ecto). CFSE-positive events (representing intact vesicles) were analysed for surface expression of Gr-1 and phosphatidylserine (PS; using annexin V) (upper lane, left to right). Controls are (lower lane, left to right): annexin buffer alone, CFSE threshold set on sonicated BM-Ecto previously stained with CFSE, BM-Ecto stained with annexin V and IgG1 isotype in PBS. FSC denotes forward scatter, SSC side scatter, respectively. Numbers indicate % positive BM-Ecto (B) Morphology of BM-Ecto (size bar 100 nm) and (C) monosodium urate (MSU) crystals (size bar 50 µm) as determined by transmission electron microscopy and light microscopy, respectively. (D) In vitro stimulation protocol. B6 peritoneal macrophages were primed with LPS for 10 h and subsequently stimulated with 100 µg/mL MSU for 4 h. BM-Ecto (1×10^8^ BM-Ecto /2×10^6^ macrophages) were given either prior (BM-Ecto+LPS>MSU) or after (LPS>BM-Ecto+MSU) LPS priming as outlined. Alternatively, PS-liposomes or control PC-liposomes were given instead of BM-Ecto. (E and G) IL-1β in cell culture supernatants determined by ELISA. n=4 per group. (F and H) Cell extracts (XT) and supernatants (SN) were analysed for the presence of NALP3, pro-IL-1β, IL-1β and active caspase 1 (p20 and p10) by western blot. Data in A, F and H are representative of three independent experiments. ***p<0.001. Mean±SEM is shown.

We first set out to find a source of murine ectosomes using B6 wild type (WT) bone marrow cells as a close approximation of neutrophils. BM-Ecto were isolated by sequentially centrifuging supernatants of bone marrow cells stimulated with fMLP. Flow cytometric analysis identified intact vesicles that were able to retain CFSE. Further characterisation revealed the expression of the neutrophil marker Gr-1 and surface exposure of PS (assessed by annexin V staining) on approximately 70–80% of intact BM-Ecto (figure 1A). Consistent with data from human neutrophil-derived ectosomes,^9^ electron microscopy of BM-Ecto preparations revealed round shaped vesicles with a size of 50–500 nm (figure 1B).

We next asked whether BM-Ecto suppress MSU-induced inflammasome activation of resident peritoneal macrophages in vitro. Inflammasome activation in vitro is a two-step process that requires priming with a TLR ligand (LPS)^12^ prior to stimulation with a specific inflammasome activator (MSU) (figure 1C). Whereas upregulation of pro-IL-1β and NALP3 components in cells is the measure of successful priming, release of mature IL-1β along with the caspase 1 subunits p20 or p10 defines efficient inflammasome stimulation.^1^ ^13^ ^14^ To determine whether BM-Ecto interfere with inflammasome activation, BM-Ecto were given to macrophages either prior to LPS priming or prior to stimulation with MSU (figure 1D) once all LPS priming events (pro-ILβ and NALP3 upregulation) had taken place.

The release of IL-1β into culture supernatants was significantly suppressed when macrophages received BM-Ecto either prior to LPS priming or MSU stimulation (figure 1E), indicating that BM-Ecto acted on both phases of inflammasome activation. Immunoblots of cell extracts (figure 1F) and cell culture supernatants (figure 1F) revealed that incubation of macrophages with BM-Ecto prior to LPS impaired efficient priming (less NALP3 and pro-IL-1β expression) and subsequently rendered macrophages unresponsive to MSU stimulation (no caspase 1 p20/10 and IL-1β release). The addition of BM-Ecto after LPS priming resulted in partial suppression of the MSU response (no caspase 1 p20 and less IL-1β), consistent with data obtained by ELISA (figure 1E). These results indicated that BM-Ecto contain inflammasome activation in vitro by suppressing LPS priming and MSU stimulation independently of each other.

The anti-inflammatory effects of Ecto have been attributed to their surface expression of PS^15^ analogous to what has been reported for apoptotic cells.^4^ ^5^ ^7^ We therefore asked whether the in vitro effects of BM-Ecto could be reproduced by size-matched liposomes expressing PS. Following our in vitro stimulation protocol (figure 1D) macrophages were treated with equal amounts (approximately 1×10^8^) of PS or control PC liposomes. While PS liposomes interfered with LPS priming (figure 1G, H, cell extracts) and MSU stimulation (figure 1G, H, cell supernatants), PC liposomes failed to attenuate the inflammasome response at any level. These results suggested that PS is involved in the inhibition of inflammasome activation by BM-Ecto.

### C5a and MSU-induced inflammation

Inflammasome activation in macrophages is functionally limited by low expression of pro-IL-1β and therefore requires some form of priming.^1^ ^12^ During gout, however, activation of the NLRP3 inflammasome occurs in a sterile environment and has been shown to be independent of Toll-like-receptor 4.^16^ Therefore the use of LPS as a priming agent in vitro does not accurately reflect what occurs in vivo. MSU crystals are known to activate complement by assembling a functional C5 convertase complex at the crystal surface, which results in the generation of active C5a.^17^ C5a fragments have been reported to activate NFκB.^18–20^ Given that the injection of MSU alone can trigger IL-1β release,^1^ ^21^ we hypothesised that C5a generated by MSU is responsible for inflammasome priming in vivo.

**Figure 2:**
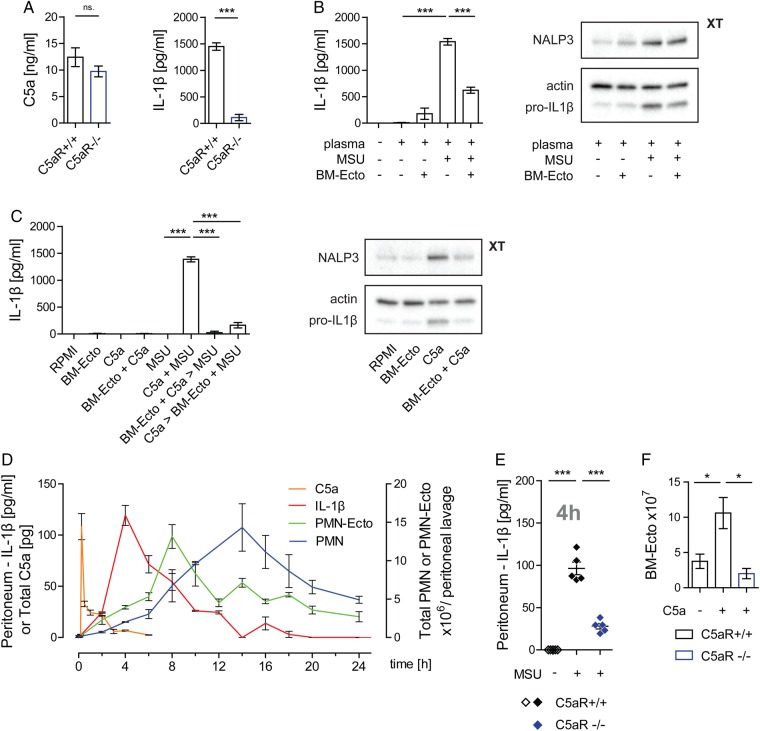
Role of C5a in monosodium urate (MSU)-induced inflammation. (A) Inflammasome activation by MSU is dependent on C5a in vitro. Generation of C5a and IL-1β release from C5aR^+/+^ or C5aR^−/−^ peritoneal macrophages stimulated with 100 µg/mL MSU in the presence of 25% plasma for 14 h in vitro. n=6 per group pooled from two independent experiments. (B and C) BM-Ecto inhibit C5a-mediated inflammasome activation in vitro. (B) BM-Ecto were given to B6 macrophages prior to stimulation with 100 µg/mL MSU in the presence of 25% plasma for 14 h or to (C) macrophages primed with 10 ng/mL recombinant mouse C5a for 10 h and subsequently stimulated with 100 µg/mL MSU for 4 h. IL-1β release, pro-IL-1β and NALP3 expression were analysed as in figure 1. n=6 per group pooled from two independent experiments (ELISA), western blots representative of two independent experiments. (D) Kinetics of MSU-induced peritonitis. B6 mice received an intraperitoneal injection of 3 mg MSU. At the indicated time points thereafter, peritonea were lavaged and infiltrating cells phenotyped by flow cytometry PMN were identified as CD45^+^, CD11b^+^, Ly6C^+^, Ly6G^+^ cells. Peritoneal concentrations of C5a and IL-1β were determined by ELISA. n=4/time point pooled from two independent experiments. (E) Inflammasome activation is C5a-dependent in vivo. Concentration of IL-1β in the peritoneal lavage fluid of C5aR^+/+^ and C5aR^−/−^ mice 4 h after injection of 3 mg MSU. n=5 pooled from two independent experiments. (F) Release of BM-Ecto requires the C5aR. 1×10^7^ bone marrow cells were stimulated with 10 ng/mL recombinant mouse C5a for 30 min at 37°C. BM-Ecto were isolated from the supernatant as indicated in Methods. n=5 pooled from two independent experiments. *p<0.05, ***p<0.001. Mean±SEM is shown.

MSU crystals generated C5a in the presence of plasma, which in turn primed the inflammasome via the C5aR leading to the release of IL-1β upon MSU stimulation of macrophages in vitro (figure 2A). BM-Ecto were capable of suppressing IL-1β release from macrophages stimulated by MSU in the presence of plasma (figure 2B) and macrophages primed with C5a and subsequently stimulated with MSU (figure 2C). These results suggested that C5a is essential for inflammasome activation by MSU and that BM-Ecto inhibit C5a-mediated inflammasome priming.

To study inflammasome activation in vivo, we next adopted a murine model of MSU-induced peritonitis.^21^ We first analysed the course of the inflammatory response following intraperitoneal injection of MSU. There was an almost immediate and steep rise of C5a 15 min after introducing MSU into the peritoneum (figure 2D). The generation of C5a was followed by the release of IL-1β in the peritoneum peaking 4 h after MSU injection (figure 2D). The release of C5a and IL-1β triggered a rise in blood neutrophils (not shown), which then infiltrated the peritoneal compartment reaching a maximum 14 h after stimulation (figure 2D). In accordance with our in vitro results, the release of IL-1β in response to MSU was significantly impaired in C5aR deficient mice (figure 2E).

We next determined whether Ecto are released by infiltrating neutrophils during MSU-induced peritonitis. At various time points following MSU stimulation, Ecto were isolated from peritoneal lavages by sequential centrifugation. Using flow cytometry, surface staining with annexin V and anti-Gr-1 identified them as PS-positive microvesicles of neutrophil origin (see online supplementary figure S1B). These neutrophil ectosomes (PMN-Ecto) were found to be present in significant numbers, reaching up to 1.5×10^7^ in the peritoneum 8 h after MSU stimulation (figure 2D). The kinetics of PMN-Ecto suggested that their release is an early event of neutrophil activation. Given that bone marrow cells release BM-Ecto in response to C5a, complement activation is the likely trigger for ectosome release in gout (figure 2F).

### Administration of Ecto attenuates MSU-induced inflammation in vivo

To determine whether Ecto have anti-inflammatory properties in vivo, the peritoneal compartment was pretreated with 2×10^7^ Ecto 2 h prior to the intraperitoneal injection of MSU. The Ecto used to precondition the peritoneum were either BM-Ecto isolated from ex vivo stimulated bone marrow cells or PMN-Ecto isolated from the lavage fluid of MSU-inflamed peritonea. The quantity (2×10^7^) of preinjected BM-Ecto and PMN-Ecto corresponded to the maximum number of PMN-Ecto recovered during MSU-induced peritoneal inflammation (figure 2D).

**Figure 3:**
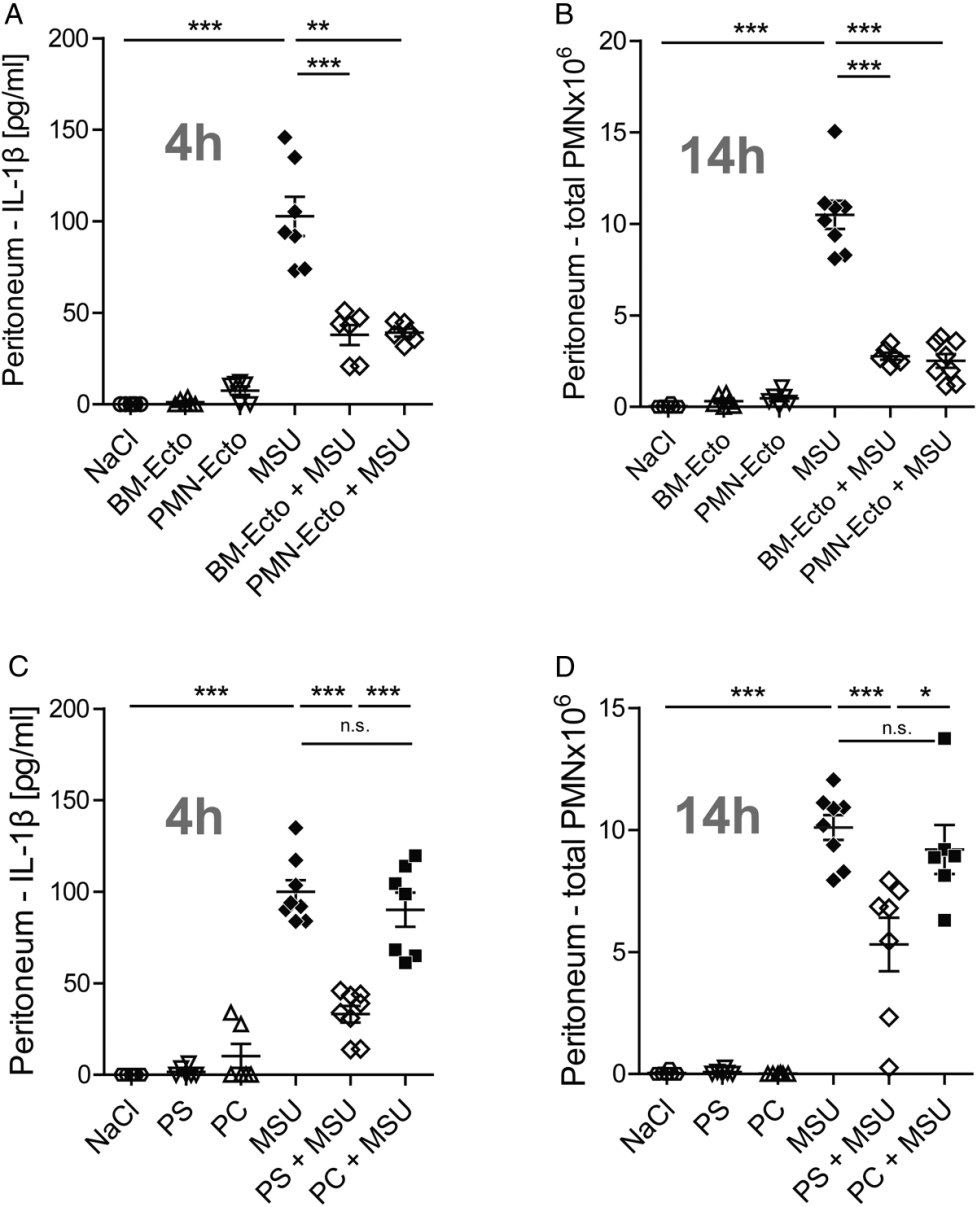
Administration of ectosomes attenuates monosodium urate (MSU)-driven peritoneal inflammation. B6 mice were injected intraperitoneally with 3 mg MSU. Where indicated, mice were preinjected with 2×10^7^ BM-Ecto or PMN-Ecto intraperitoneally 2 h prior to MSU stimulation. Alternatively, mice were preinjected with 75 nM (approximately 2×10^7^) of phosphatidylserine (PS)-liposomes or phosphatidylcholine (PC)-liposomes. Control groups received BM-/PMN-Ecto or PS-/PC-liposome injections intraperitoneally followed by NaCl instead of MSU. (A and C) IL-1β in peritoneal lavage fluid was determined by ELISA 4 h after MSU stimulation. (B and D) The number of infiltrating PMN into the peritoneum 14 h after MSU stimulation was determined as indicated in figure 2D. n=6–8 per group pooled from at least three independent experiments, *p<0.05, **p<0.01, ***p<0.001. Mean±SEM is shown.

Pretreatment with PMN-Ecto or BM-Ecto resulted in a twofold suppression of IL-1β release in response to MSU (figure 3A) and subsequently a threefold decrease in the number of infiltrating neutrophils into the peritoneal compartment 14 h after stimulation (figure 3B). Taken together, these results indicated that Ecto from two independent sources can suppress inflammation induced by MSU.

### The in vivo effects of Ecto can be mimicked by liposomes expressing PS

To confirm that PS liposomes can act as a surrogate for Ecto in vivo, approximately 2×10^7^ PS or control PC liposomes were injected intraperitoneally instead of Ecto 2 h prior to stimulation with MSU. Pretreatment with PS liposomes resulted in suppression of IL-1β (figure 3C) and neutrophil influx into the peritoneum (figure 3D) in response to MSU. Injection of control PC liposomes failed to achieve an anti-inflammatory effect.

### Suppression by BM-Ecto is MerTK-dependent in vivo

To directly test the involvement of PS in Ecto-mediated immunosuppression, we next applied our model to mice lacking the MerTK receptor (*MerTK^−/−^*) which is known to bind PS and relay its signal by inducing SOCS3.^7^

**Figure 4:**
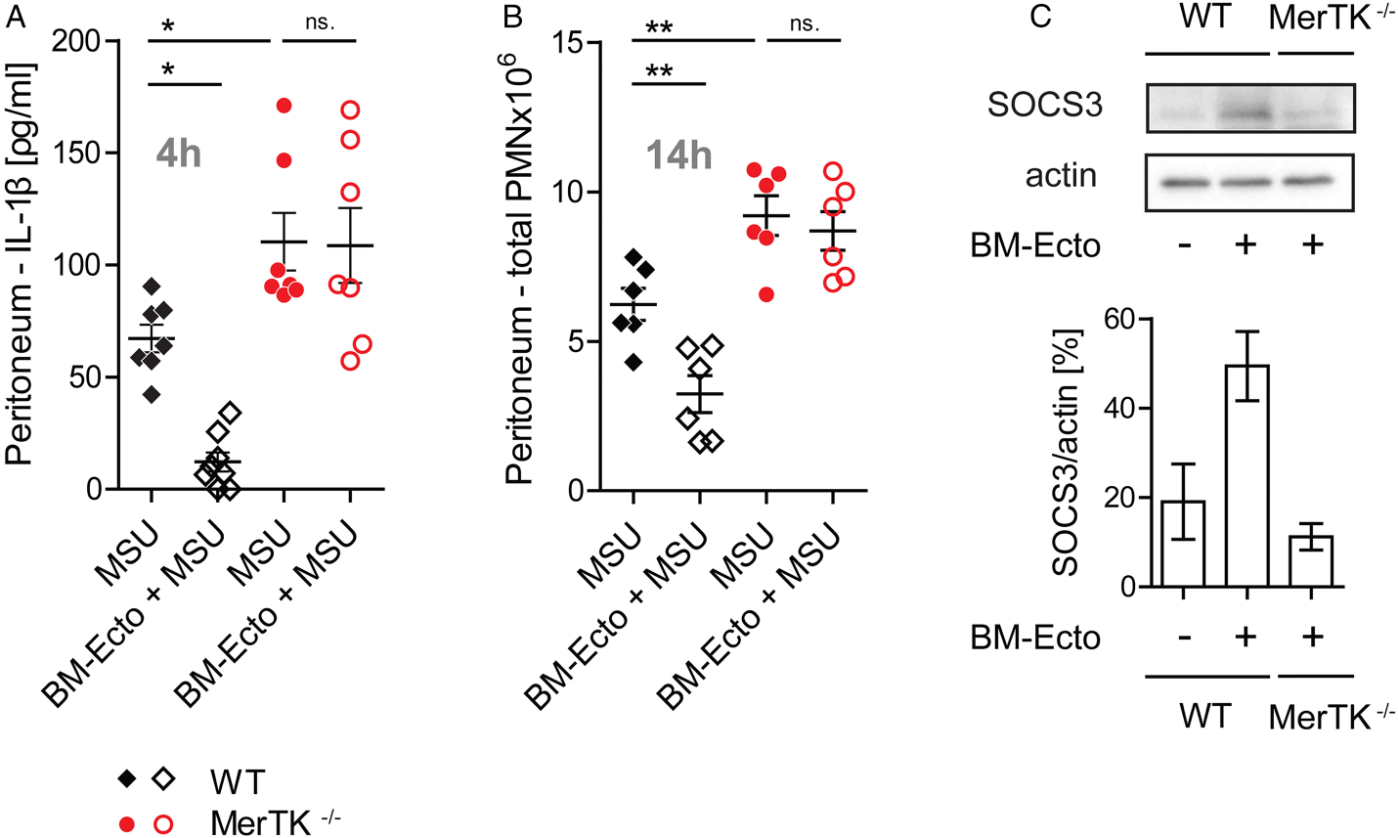
Ecto-mediated immunosuppression requires MerTK in vivo. B6/129S (wild type, WT) or *MerTK^−/−^* mice were injected intraperitoneally with 3 mg monosodium urate (MSU). Where indicated, mice were preinjected with 2×10^7^ BM-Ecto intraperitoneally 2 h prior to MSU stimulation. (A) IL-1β was determined 4 h after MSU stimulation in the peritoneal lavage fluid. (B) The number of infiltrating PMN into the peritoneum 14 h after MSU stimulation was determined as in figure 2D. n=6–8 per group, pooled from at least three independent experiments. (C) Suppressor of cytokine signalling (SOCS3) expression in peritoneal macrophages of B6/129S (WT) and *MerTK^−/−^* mice determined by immunoblot 4 h after intraperitoneal BM-Ecto injection. Expression of SOCS3 in arbitrary units of band intensity normalised to actin. n=2 per group pooled from two independent experiments. *p<0.05, **p<0.05. Mean±SEM is shown. n.s., not significant.

The peritonea of *MerTK^−/−^* and control B6129S (WT) mice were pretreated with BM-Ecto prior to MSU stimulation. Whereas pretreatment of WT mice with BM-Ecto led to suppression of IL-1β release (figure 4A) and neutrophil influx (figure 4B) in the peritoneum, the anti-inflammatory effects of BM-Ecto were absent in *MerTK^−/−^* mice. Furthermore, 4 h after intraperitoneal BM-Ecto injection, we observed a MerTK-dependent induction of SOCS3 in peritoneal macrophages (figure 4C). Of note, inflammation induced by MSU in *MerTK^−/−^* mice was significantly higher compared with background-matched WT mice (figure 4A, B), suggesting that endogenously released PMN-Ecto limit the magnitude of MSU-induced inflammation via MerTK in WT mice.

### BM-Ecto induce the release of TGFβ

**Figure 5:**
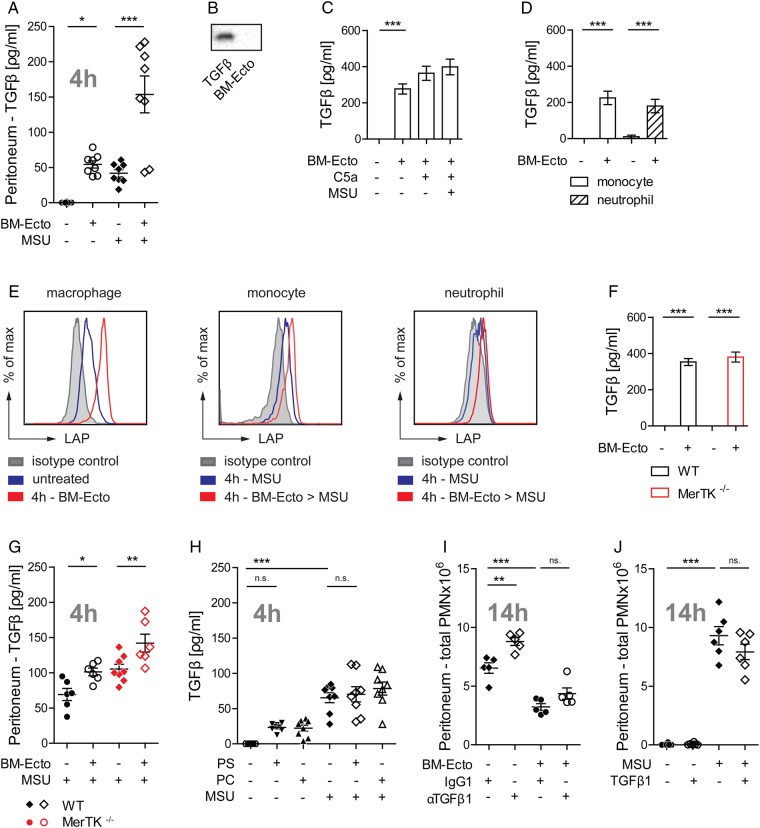
Ecto induce the release of TGF-β independent of MerTK. (A) TGFβ concentration in peritoneal lavage fluids of B6 mice treated as outlined in figure 3 was determined by ELISA. n=6–8 pooled from three independent experiments. (B) Ecto are not the source of TGFβ in vivo. BM-Ecto lysates were assessed for TGFβ content by immunoblot. Recombinant mouse TGFβ was used as control. (C–E) Cellular source of TGFβ. Release of TGFβ by B6 (C) resident peritoneal macrophages or (D) monocytes and neutrophils isolated from monosodium urate (MSU)-inflamed peritonea following treatment with C5a, MSU and/or BM-Ecto in vitro as outlined in figure 2C. (E) Expression of latency associated peptide (LAP) on B6 macrophages, monocytes and neutrophils isolated either 4 h after intraperitoneal injection of BM-Ecto or 4 h after intraperitoneal injection of MSU with BM-Ecto pretreatment. Controls received NaCl intraperitoneally (untreated). (F–H) TGFβ release is independent of MerTK. (F) The release of TGFβ by wild type (WT) and *MerTK^−/^*^−^ macrophages treated with BM-Ecto in vitro. n=6/group. TGFβ in peritoneal lavage fluids of (G) B6/129S (WT) and MerTK^−/−^ mice treated with BM-Ecto or (H) B6 mice treated with liposomes as outlined in figures 3 and 4 ,respectively. n=6–8/group. (I and J) Effect of TGFβ in vivo. (I) The effect of neutralising anti-TGFβ1 antibodies on peritoneal PMN influx using 100 µg anti-TGFβ1 injected intraperitoneally 15 min prior to MSU or 15 min prior to BM-Ecto pretreatment. (J) The effect of 1 µg recombinant mouse TGFβ1 injected intraperitoneally instead of BM-Ecto prior to MSU stimulation. *p<0.05, **p<0.01, ***p<0.001. Mean±SEM is shown. n.s., not significant.

It has been suggested that TGFβ participates in the resolution of gout in its late stages.^22^ Therefore, we next asked whether Ecto induce the release of TGFβ in vivo. Injection of BM-Ecto induced the release of TGFβ in the peritoneum (figure 5A). The release of TGFβ was further enhanced when BM-Ecto primed mice received MSU (figure 5A). Since BM-Ecto themselves were not the source of TGFβ (figure 5B) and the amount of TGFβ released in vitro by peritoneal macrophages remained the same regardless of inflammasome activation (figure 5C), we hypothesised that the additive effect of MSU and BM-Ecto on TGFβ release could be due to infiltrating cells responding to BM-Ecto. Indeed, monocytes and neutrophils isolated from MSU inflamed peritonea were able to release TGFβ in response to BM-Ecto in vitro (figure 5D). To determine which of these cells contributes the most to the TGFβ pool in vivo, resident macrophages, infiltrating monocytes and neutrophils from BM-Ecto-treated peritonea were stained for latency associated peptide (LAP). LAP is part of latent TGFβ and remains tethered to the surface of macrophages^23^ and monocytes^24^ once TGFβ is released and cleaved into its active form. Whereas expression of LAP in naive resident peritoneal macrophages (F480^+^, CD115^+^, Ly6C^−^) was low, LAP progressively increased over time after intraperitoneal injection of BM-Ecto, indicating continuous TGFβ release (figure 5E). Monocytes and neutrophils are not present in untreated peritonea. Upon intraperitoneal MSU stimulation, however, infiltrating monocytes (Ly6C^+^, F480^−^, Ly6G^−^) and, to a lesser extent, neutrophils (Ly6C^+^, F480^−^, Ly6G^+^) upregulated LAP when the peritoneum was pretreated with BM-Ecto prior to MSU (figure 5E).

### Ecto suppress MSU-induced peritonitis independent of TGFβ

We next asked whether TGFβ was necessary for the suppressive effects of Ecto. In vitro, TGFβ release was independent of MerTK (figure 5F). In vivo, significant increases in TGFβ were consistently measured in the peritonea of WT and *MerTK^−/−^* mice pretreated with BM-Ecto (figure 5G). Moreover, PS liposomes did not induce TGFβ release in vivo (figure 5H). Since PS liposomes attenuated the response to MSU (figure 3C, D) without inducing TGFβ and BM-Ecto failed to inhibit inflammation in *MerTK^−/−^* mice (figure 4A, B) despite releasing TGFβ (figure 5G), TGFβ did not seem to be necessary for BM-Ecto-mediated resolution of acute gouty inflammation. To confirm that the effect of BM-Ecto was independent of TGFβ, neutralising anti-TGFβ1 antibody was given intraperitoneally 30 min prior to BM-Ecto. In the presence of TGFβ1-blocking antibody, BM-Ecto still retained their capacity to suppress inflammation (figure 5I). Although the blocking of TGFβ1 slightly increased neutrophil influx in response to MSU suggesting that TGFβ may play a role (figure 5I), the injection of recombinant mouse TGFβ1 instead of BM-Ecto had no effect (figure 5J).

Taken together, these data suggested that Ecto inhibit the acute inflammatory response to MSU predominantly via the PS-MerTK pathway rather than TGFβ. Although Ecto induced the release of TGFβ by macrophages and monocytes, MerTK alone was necessary and sufficient for Ecto to suppress inflammasome activation in vivo.

### PMN-Ecto are present in synovial exudates during gouty inflammation in humans

**Figure 6:**
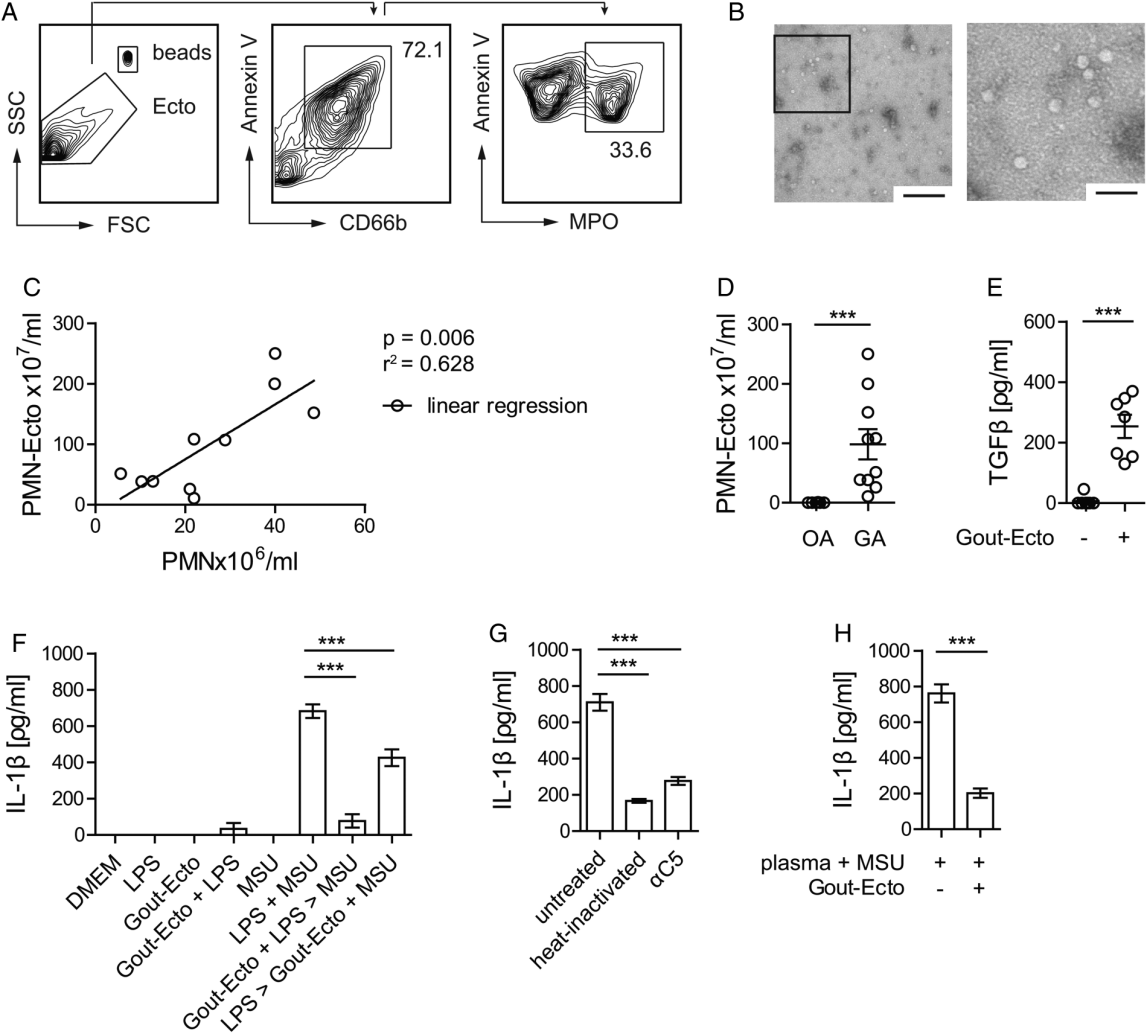
PMN-Ecto present in gout exudates in humans have immunosuppressive properties. PMN-Ecto were isolated from joint aspirates of patients undergoing a gout attack. (A) Ecto were isolated from joint aspirates as indicated in methods and characterised and counted by flow cytometry. Annexin V, anti-CD66b and anti-myeloperoxidase (MPO) antibodies identified them to be of neutrophil origin. Counting was performed using microbeads. (B) Transmission electron microscopy of PMN-Ecto. Size bar 1 µm (left) and 100 nm (right). (C) Correlation between the number of infiltrating PMN and PMN-Ecto found in gout exudates. Each dot represents a single patient. (D) Concentration of PMN-Ecto in gout (gouty arthritis, GA) and osteoarthritis (OA) exudates. (E–H) PMN-Ecto isolated from joint aspirates are functional in vitro. (E) Release of TGFβ by human monocyte-derived macrophages (HMDM) treated with Gout-Ecto (1×10^8^ Gout-Ecto/2×10^6^ macrophages). n=7 pooled from two independent experiments. (F) Suppression of IL-1β release by HMDM treated with Gout-Ecto following the in vitro protocol outlined in figure 1D. (G) IL-1β release upon 14 h stimulation of HMDM with 100 µg/mL monosodium urate (MSU) in 25% human plasma (untreated), heat inactivated plasma (heat inactivated) or C5-blocked plasma (αC5). (F and G) n=6 pooled from three independent experiments. (H) Suppression of IL-1β release by HMDM treated with Gout-Ecto and stimulated as outlined in (G). ***p<0.001. Mean±SEM is shown.

Lastly, we sequentially centrifuged synovial exudates of patients with GA and control patients with OA to verify the presence of microvesicles during gouty attacks in humans. Arthrocentesis was performed within 1 day after the onset of symptoms. Using flow cytometry, we found annexin V-positive vesicles expressing the granulocyte marker CD66b and the neutrophil-specific enzyme myeloperoxidase (figure 6A). Electron micrographs (figure 6B) of exudates confirmed the presence of intact vesicles that were approximately 50 nm in size.

The amount of PMN-Ecto isolated from gout exudates correlated with the number of infiltrating PMN (figure 6C) and GA exudates had significantly higher numbers of PMN-Ecto compared to OA exudates (figure 6D). Recovery of neutrophil-derived ectosomes from synovial exudates of patients during gouty inflammation suggested that PMN-Ecto release occurs and is relevant in vivo.

In vitro, PMN-Ecto from gout exudates (Gout-Ecto) were able to induce the release of TGFβ from human monocyte-derived macrophages (figure 6E) and inhibited the release of IL-1β by macrophages (figure 6F) treated as outlined in figure 1D. Furthermore, in the presence of human plasma, MSU crystals were able to simultaneously prime and stimulate the inflammasome. Consistently, MSU failed to prime the inflammasome in the presence of heat inactivated or C5-blocked plasma, confirming that priming is C5-dependent (figure 6G). Finally, Gout-Ecto suppressed IL-1β release in macrophages stimulated by MSU in the presence of human plasma (figure 6H).

## Discussion

The major findings of the present study are related to C5a and PMN-Ecto release in gout. C5a generated by MSU is responsible for priming the inflammasome and consequently for the release of IL-1β. Furthermore, C5a induces PMN-Ecto release by infiltrating neutrophils and these ectosomes in turn limit inflammasome priming. Ectosomes achieve their anti-inflammatory effect by engaging the MerTK receptor. The regulation induced by PMN-Ecto starts almost immediately after the influx of cells into the peritoneum, indicating that the control of inflammation starts much earlier than presumed until now. Interestingly, the very cells that are responsible for acute inflammation (ie, neutrophils), act also as its regulator due to the shedding of ectosomes.

The release of PMN-Ecto is an early phenomenon of neutrophil activation.^9^ In our gout model, PMN-Ecto were released as early as 2 h after intraperitoneal injection of MSU and their concentration peaked at 8 h. To analyse their effect on gouty inflammation, ectosomes were given intraperitoneally prior to MSU injection. The number of ectosomes injected was physiological and the amount was set at the maximum number of PMN-Ecto recovered from the peritoneum after MSU stimulation. Since ectosomes bind back to cells^9^ and are continuously cleared,^25^ the recovery of 1.5×10^7^ PMN-Ecto is likely an underestimation of the total amount of ectosomes shed during the course of MSU peritonitis.

Previous studies have already suggested that PS expressed on apoptotic cells^3–5^ ^7^ and ectosomes^26^ contribute to the resolution of inflammation. PS has been shown to mediate anti-inflammatory signals via the MerTK receptor.^5^ ^7^ MerTK activation leads to induction of SOCS3^6^ and in turn to suppression of TLR-induced cytokine release.^5^ ^7^ ^15^ In accordance with these studies, we could confirm that PS-expressing ectosomes suppress LPS priming of the NLRP3 inflammasome in vitro. LPS priming, however, is not required for the inflammatory response to MSU in vivo and is therefore merely used to model inflammasome activation in vitro.^16^ In addition to activating the inflammasome, MSU crystals activate the complement cascade by assembling a C5 convertase on their surface.^17^ ^27^ We could confirm that C5a generated by MSU is the main inflammasome priming agent in gout in vivo.^28^ The anti-inflammatory effect of PMN-Ecto was therefore not limited to the inhibition of TLR stimuli, but extended to C5a and C5aR signalling as well. Furthermore, C5a was able to prime the inflammasome and limit its activation by inducing the release of PMN-Ecto, thus forming the basis of an autoregulatory negative feedback loop in gout.^9^ This mechanism may apply to other crystal arthropathies such as those caused by calcium pyrophosphate dihydrate and hydroxyapatite crystals, since Ecto suppress calcium pyrophosphate dihydrate and hydroxyapatite-induced IL-1β release as well (see online supplementary figure S1C).

Whereas studies using liposomes suggested that PS-positive vesicles could downregulate inflammation, involvement of PS was confirmed in mice deficient for the PS receptor MerTK. Furthermore, the higher degree of inflammation in *MerTK^−/−^* compared with WT mice suggested that PS expressed on Ecto provides baseline suppression in gouty inflammation. Of note, high expression of SOCS3 in synovial tissue has been found in patients during the acute phase of gout,^8^ further supporting the notion that the PS-MerTK axis plays a role in limiting gouty inflammation.

TGFβ is considered to play an active role in the resolution of gout.^8^ ^29^ ^30^ Levels of TGFβ have been found to progressively increase in synovial fluids of patients with gout after an attack, suggesting that TGFβ may be involved in late phases of resolution.^8^ ^22^ We demonstrated that ectosomes trigger the release of TGFβ in vitro and in vivo. These findings are consistent with other studies that have shown release of TGFβ by macrophages^4^ or neutrophils^3^ in response to apoptotic cells and ectosomes.^26^ In vivo, however, neither treatment with recombinant mouse TGFβ1 (figure 5J), nor the release of TGFβ by ectosomes in the absence of PS-MerTK activation (figure 5G) was sufficient to control the acute inflammatory response to MSU. Furthermore, PS-liposomes alone could reproduce the ectosome effect without inducing the release of TGFβ (figure 3C-D, figure 5H) and TGFβ blocking did not compromise the ability of BM-Ecto to suppress inflammation (figure 5I). These findings argue against a role for TGFβ in the early phase of gout. Given that patients with gout with TGFβ polymorphisms frequently progress to more advanced disease states, TGFβ may play a role in chronic gout and affect the rate of progression rather than resolution of acute attacks.^31^ Aside from TGFβ, IL-10 and IL-1Ra have also been associated with anti-inflammatory effects, but they were not induced by ectosomes in the MSU peritonitis model (see online supplementary figure S1D–F).^32–34^

Recently, the resolution of gout has been associated with another phenomenon of neutrophil activation, the release of neutrophil extracellular traps (NETs).^35^ NETs were shown to degrade proinflammatory chemokines by clustering serine proteases such as proteinase 3 and neutrophil elastase. In our model of acute gout we could not measure significant increase of cell-free DNA or NETs (see online supplementary figure S1G) and the treatment with DNAse I did not significantly alter the outcome of MSU-induced inflammation (see online supplementary figure S1H). Although NETs did not play a role in this model, the degradation of chemokines by proteases may still be relevant, since PMN-Ecto actively recruit proteinase 3and neutrophil elastase from fluid phase to their surface.^9^ ^36^ PMN-Ecto may not only suppress IL-1β release upon MSU stimulation, but potentially also degrade IL-1β once it has been released. This could possibly explain the higher efficacy of PMN-Ecto compared with liposomes, which carry no enzymes (figure 3B, D).

A limitation of our study was the exclusive use of the MSU peritonitis model. It would be of interest to assess whether ectosomes elicit similar responses in alternate models of gout, such as the intra-articular or air-pouch model, which are known to have various degrees of NLRP3, ASC and caspase 1 involvement in IL-1β release.^37^

The motivation behind this work was to better understand the self-limiting nature of gout. This study supports the notion that resolution of gout is initiated with the release of PMN-Ecto early on during neutrophil activation. In a broader context, PMN-Ecto release may limit excessive inflammation in response to exogenous and endogenous danger signals and their pathophysiological relevance likely extends to conditions other than gout.^38^

## Supplementary Material

Web supplement
