# Supplementary material for: Neutrophil microvesicles resolve gout by inhibiting C5a-mediated priming of the inflammasome
Source: Ann Rheum Dis. 2015 Aug 5;75(6):1236–45. doi: 10.1136/annrheumdis-2015-207338 (PMC4893114; doi:10.1136/annrheumdis-2015-207338)
Supplement: Web supplement [file annrheumdis-2015-207338-s1.pdf]

SUPPLEMENTARY MATERIALS AND METHODS

Mice. C57BL/6 (B6) and Balb/c mice were bred at the institution's animal facility or purchased from Charles River (Sulzfeld, Germany). B6;129-Mertktm1Grl/J (*MerTK*$^{-/-}$) and B6;129SF2/J wild-type mice were from The Jackson Laboratories (Bar Harbour, USA). C.129S4(B6)-C5ar1tm1Cge/J (C5aR$^{-/-}$) mice on a Balb/c background were a generous gift from Prof. van den Broek (Institute of Experimental Immunology, University of Zürich, Switzerland). All mice were housed in a specific pathogen-free environment and were used between 6 and 12 weeks of age. Animal care and experimentation were performed in accordance with national guidelines (Federal Veterinary Office) and had been approved by the local government authorities.

Reagents and Antibodies. Fluorochrome-conjugated antibodies against CD11b (clone M1/70), Gr-1 (RB6-8C5), Ly6C (AL-21), Ly6G (1A8), CD115 (AFS98), F4/80 (BM8), CD45 (30F11), CD66b (G10F5) and LAP were from Biolegend (San Diego, USA). Purified anti-CD16/32 (2.4G2) was from BD Pharmingen (Allschwill, Switzerland), annexinV-APC from Immunotools (Friesoyte, Germany) and anti-MPO (MPO455-8E6) from eBiosciences (Vienna, Austria). 7-AAD and CFSE were from Invitrogen (Zug, Switzerland), monosodium urate and fMLP from Sigma (Schnelldorf, Germany). Anti-IL1β (AF-401-NA) and anti-TGFβ (9016) were from R&D Systems (Abingdon, UK), anti-NALP3 (Cryo-2) and anti-caspase 1 p20 (Casper-1) from Adipogen (Liestal, Switzerland), anti-actin (C4), anti-TGFβ (H-112), anti-SOCS3 (H-103) and HRP conjugated anti-mouse (sc-2005), -rabbit (sc-2004) and -goat (sc-2020) antibodies were from Santa Cruz Biotechnology (Muttenz, Switzerland), anti-MPO (2D4) was from Abcam, (Cambridge, UK). Anti-C5 monoclonal antibody (eculizumab®) was from Alexion Pharmaceuticals (Lausanne, Switzerland).

Generation of crystals. MSU crystals were prepared by dissolving 0.5g of MSU in 90ml de-ionized H_20 and 600µl of 5M NaOH. This MSU solution was heated to 90°C, passed across a 0.2µm filter and allowed to precipitate into crystals over-night at room temperature. The slurry was subsequently washed twice with ethanol, once with acetone and then allowed to evaporate leaving crystals behind. Crystals were stored at -20°C and dissolved in 0.9% NaCl at 6mg/ml prior to use. Size of MSU crystal was verified by transmission light microscopy (Olympus, IX50) and acquired by Cell Sense Standard software. Calcium Pyrophosphate Dihydrate (CPPD) crystals (5-20µ in size) and Hydroxyapatite (HA) crystals were from Invivogen and Sigma, respectively. All crystals were prepared in sterile conditions with sterile filtered solutions and verified to be endotoxin-free (<0.05 IE/ml) by a Limulus amebocyte cell lysate assay (Ph Eur 2.6.14, Endosafe PTS reader PHM-Q05).

Generation of PS/PC liposomes. Unilamelar liposomes, composed of either L-α-phosphatidylcholine (PC, derived from bovine liver) alone or PC together with equimolar concentrations of L-α-phosphaditylserine (PS, derived from porcine brain, both Avanti Polar Lipids, Alabaster, USA) were prepared according to the repeated freeze-thawing method [1]. Final liposome concentration was adjusted to 16 mM as measured by a standard phosphate assay. This protocol yielded liposomes of 100-200 nm diameter as determined by electron microscopy and zetasizer (Delsa^{TM} NanoC).

Transmission electron microscopy. Microvesicles were fixed in 1% glutaraldehyde (final concentration) for 1 min at room temperature and then adsorbed to parlodion-coated copper grids. After washing, samples were stained with 2% uranylacetate before being observed in a Philips Morgani 268 D transmission electron microscope operated at 80 kV.

Isolation and culture of human macrophages. Human monocyte-derived macrophages were isolated from buffy coats of healthy blood donors as described [2]. Briefly, buffy coats were diluted 1/1(vol/vol) in HBSS, layered over Ficol-Histopaque (Sigma, St Louis, USA) and centrifuged 350g/25min/25°C with no breaks. PBMC were collected at the ficol gradient interphase, washed with PBS-2mM EDTA and MACS buffer (Miltenyi, Bergisch Gladbach, Germany). Monocytes were enriched by anti-CD14 magnetic beads (Miltenyi) according to manufacturer's instructions and allowed to mature into macrophages in tissue culture plates over 6-7 days in DMEM 10% heat-inactivated human serum.

Complement assays. C5a measurement was done by ELISA using a rat anti-mouse C5a IgG capture antibody and a biotinylated rat anti-mouse C5a detection antibody (both used at 5 µg/ml) in PBS-10% FCS assay diluent. C5a was revealed with SA-HRP (all BD). Recombinant mouse C5a (BD) was used both as ELISA standard and priming agent in vitro at 10ng/ml. For generation of mouse and human plasma, blood was anticoagulated with refludan (Celgene, Boudry, Switzerland) at final concentration of 2.5µg/ml. Human plasma was heat inactivated at 56°C for 30min. C5 in plasma was inhibited with 50µg/ml anti-C5 for 30min at 4°C.

Neutrophil Extracellular Traps (NETs) assay. The release of NETs in MSU induced peritonits was analyzed by measuring the concentration of cell free double-stranded DNA (dsDNA) and DNA-MPO complexes (representing NETs) in peritoneal lavage fluids. dsDNA content was measured using QuantiFluor dsDNA System (Promega) kit. The concentration of DNA-MPO complexes was determined by a sandwich ELISA method using anti-MPO antibodies (Abcam Cambridge, UK) to capture mouse MPO and Cell Death Detection Elisa ^{PLUS} (Roche) to measure DNA as previously described [3]. To determine the effect of NETs on the magnitude of MSU induced inflammation, the peritoneal compartment was pre-injected with 100ug of DNAse (Sigma) 15min prior to MSU stimulation.

Patient samples. Synovial arthrocenthesis samples were obtained from patients included in the Basel COUGAR cohort study. The study had been approved by the local ethics committee. All patients had given written informed consent prior to inclusion in the study.

Statistical analysis. All data are presented as mean ± SEM. Comparisons between treatment groups were performed using one-way analysis of variance (ANOVA) with Bonferroni's multiple comparison post-testing or Student's t-test (Prism version 6; GraphPad Software, La Jolla, USA). Significance was set at $P < 0.05$.

SUPPLEMENTARY FIGURE

Figure S1. Supplementary figure

(**A**) Phenotype of peritoneal exudate cells and blood leukocytes was determined by flow cytometry following surface staining with the indicated antibodies. Leukocyte populations were defined as follows: macrophages (F480^{+}, CD115^{+}, Ly6C^{-}), neutrophils (Ly6C^{+}, F480^{-}, Ly6G^{+}) and monocytes (Ly6C^{+}, F480^{-}, Ly6G^{-}). (**B**) Phenotype of PMN-Ecto. PMN-Ecto were identified as annexin V^{+}, Gr-1^{+} events by flow cytometry after sequential centrifugation of peritoneal lavage fluid. Counting of PMN-Ecto was carried out with beads as indicated in Methods. (**C**) The effect of BM-Ecto on IL-1β release by B6 macrophages stimulated with 100µg/ml calcium pyrophosphate dihydrate (CPPD) or hydroxyapatite (HA) crystals in the presence of 25% plasma for 14h. n=4 per group pooled from two independent experiments. (**D**-**F**) IL-10 and IL-1Ra concentration in peritoneal lavage fluids from experiments in Figure 2D (**D**) and Figure 3A (**E-F**). (**G**) Concentration of dsDNA and NETs, defined as DNA-MPO complexes, in peritoneal lavage fluids harvested at the indicated time points after intraperitoneal injection of 3mg MSU crystals in B6 mice. n=4/time point pooled from two independent experiments. (**H**) B6 mice were pre-injected with 100ug of DNAse 15min prior to MSU stimulation. Control groups received intraperitoneal injection with DNAse followed by NaCl instead of MSU, or NaCl alone. The number of infiltrating PMN into the peritoneum 14h after MSU stimulation was determined as indicated in Figure 2D. n=6 per group pooled from at least two independent experiments. ***P<0.001. Mean ± SEM is shown. n.s. = not significant.

Figure S1

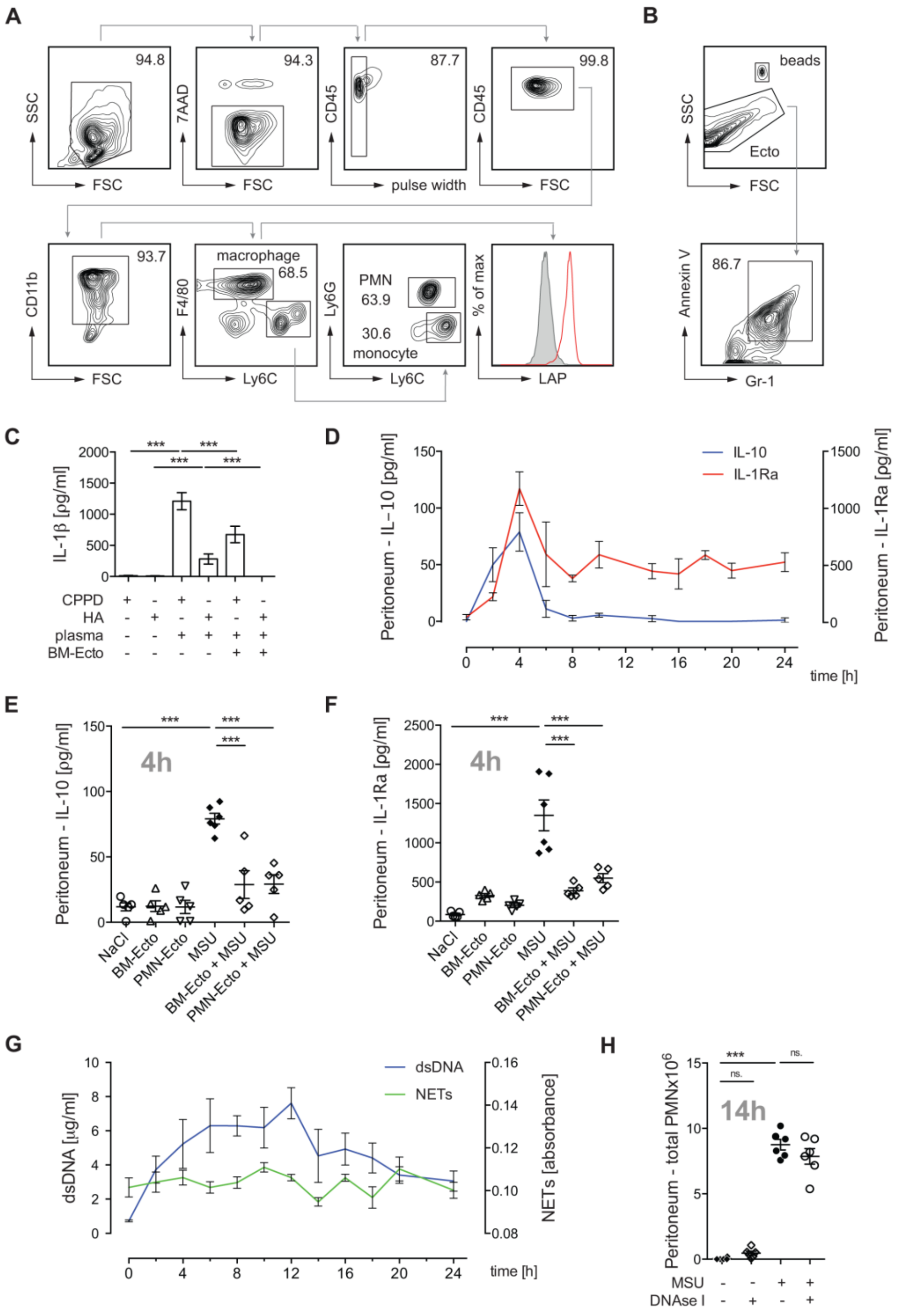

A
94.8
SSC
FSC
94.3
7AAD
FSC
87.7
CD45
pulse width
99.8
CD45
FSC
93.7
CD11b
FSC
macrophage
68.5
F4/80
Ly6C
PMN
63.9
30.6
monocyte
Ly6G
Ly6C
% of max
LAP
B
beads
Ecto
SSC
FSC
86.7
Annexin V
Gr-1
C
IL-1β [pg/ml]
CPPD
HA
plasma
BM-Ecto
D
IL-10
IL-1Ra
Peritoneum - IL-10 [pg/ml]
Peritoneum - IL-1Ra [pg/ml]
time [h]
E
4h
Peritoneum - IL-10 [pg/ml]
NaCl
BM-Ecto
PMN-Ecto
MSU
BM-Ecto + MSU
PMN-Ecto + MSU
F
4h
Peritoneum - IL-1Ra [pg/ml]
NaCl
BM-Ecto
PMN-Ecto
MSU
BM-Ecto + MSU
PMN-Ecto + MSU
G
dsDNA
NETs
dsDNA [µg/ml]
NETs [absorbance]
time [h]
H
14h
Peritoneum - total PMNx10^6
MSU
DNAse I

REFERENCES

1. Szoka F, Olson F, Heath T, Vail W, Mayhew E, Papahadjopoulos D. Preparation of unilamellar liposomes of intermediate size (0.1-0.2 mumol) by a combination of reverse phase evaporation and extrusion through polycarbonate membranes. Biochim Biophys Acta. 1980 Oct 2; 601(3):559-571.
2. Gasser O, Schifferli JA. Activated polymorphonuclear neutrophils disseminate anti-inflammatory microparticles by ectocytosis. Blood. 2004 Oct; 104(8):2543-2548.
3. Caudrillier A, Kessenbrock K, Gilliss BM, Nguyen JX, Marques MB, Monestier M, et al. Platelets induce neutrophil extracellular traps in transfusion-related acute lung injury. J Clin Invest. 2012 Jul; 122(7):2661-2671.
